# POLM inhibits porcine epidemic diarrhea virus replication by degrading multiple viral structural proteins

**DOI:** 10.1128/jvi.02278-24

**Published:** 2025-02-10

**Authors:** Xinyu Cao, Yingyu Liu, Wu Tong, Wenzhen Qin, Xinyu Yang, Hai Yu, Hao Zheng, Wen Zhang, Guangzhi Tong, Ning Kong, Tongling Shan

**Affiliations:** 1 College of Veterinary Medicine, Xinjiang Agricultural University 117840 , Urumqi, China; 2 Shanghai Veterinary Research Institute, Chinese Academy of Agricultural Sciences 118161 , Shanghai, China; 3 School of Medicine, Jiangsu University 191611 , Zhenjiang, China; 4 Jiangsu Co-Innovation Center for the Prevention and Control of Important Animal Infectious Disease and Zoonoses, Yangzhou University 38043 , Yangzhou, China; University of Kentucky College of Medicine, Lexington, Kentucky, USA

**Keywords:** POLM, PEDV, N protein, S2 protein, M protein, MARCH8, p62, autophagy

## Abstract

Porcine epidemic diarrhea, as a porcine epidemic diarrhea virus (PEDV)-induced infectious intestinal condition typified by diarrhea, emesis, dehydration, and anorexia, leads to death rates as high as 100% among suckling piglets. Given the existing commercial vaccines, it is essential to study host-virus interactions and formulate efficient anti-viral regimes. This study concerned a host factor POLM (a DNA polymerase family member) that exerts an anti-viral effect against PEDV proliferation. Our results indicated that POLM expression was increased following PEDV infection and was regulated by the transcription factor FOXA1. In addition, our findings indicated that POLM targeted and degraded PEDV structural proteins (N, S2, and M) by the autophagy pathway to inhibit PEDV proliferation. POLM could recruit the E3 ubiquitination ligase MARCH8 for N, S2, and M protein ubiquitination, which was subsequently recognized by p62, a cargo receptor, for translocation to the autophagic lysosome, therefore degrading the N, S2, and M proteins and preventing PEDV proliferation. In summary, we showed a novel therapeutic target for combating PEDV, i.e., using the POLM-MARCH8-p62-autophagosome pathway to degrade the PEDV N, S2, and M proteins.

**IMPORTANCE:**

PEDV is a coronavirus that causes high mortality in piglets, which poses significant economic damage to swine farming. During PEDV infection, the host cells may promote the natural anti-viral immune response to suppress viral replication through a variety of potential host factors. In this study, we found upregulation of a host factor POLM by FOXA1 (a transcription factor) during PEDV infection. It was indicated that POLM could be a new anti-viral protein against the PEDV replication, which interacted with MARCH8 (an E3 ubiquitin ligase) and p62 (a cargo receptor) to facilitate the PEDV N, S2, and M protein degradation via the autophagy process. Apart from elucidating a previously unidentified anti-viral function of POLM, this study also provides a novel perspective for studying host anti-viral factors that act as regulators of anti-PEDV protein degrading pathways.

## INTRODUCTION

Porcine epidemic diarrhea refers to a porcine epidemic diarrhea virus (PEDV)-induced highly infectious acute intestinal condition, which is basically typified by watery diarrhea, emesis, dehydration, and anorexia (1, 2). While all age groups can be infected and manifest a range of symptoms, the symptoms are particularly severe in piglets, with mortality rates reaching 100% (3). PEDV is a type of single-stranded, enveloped, positive-sense RNA alphacoronavirus from the *Coronaviridae* family (4, 5). Its genome has a length of about 28 kb and consists of seven open reading frames (ORFs). Among them, ORF1a and ORF1b are responsible for encoding nonstructural proteins that control genomic transcription, replication, as well as polyprotein processing. The other five ORFs encode four structural proteins, namely, spike (S), nucleocapsid (N), membrane (M), and envelope (E), alongside an accessory ORF3 protein (1, 6, 7). As a principal factor influencing viral tropism and entry, the S protein represents a crucial target of the host antibody response. The ectodomain of the S protein can be subdivided into two subunits (S1 and S2) that fulfill distinguishing functions. The N-terminal S1 subdomain realizes receptor-binding function, while the membrane-anchored C-terminal S2 subdomain facilitates fusion with the membrane (8, 9). The N protein can serve as a target for precise early detection of PEDV infection, which also offers a structural foundation for helical nucleocapsid by interacting with PEDV genomic RNA. The M protein, which is essential for viral envelope formation, interacts with the entire structural components in the virion assembly process. The E protein, which possesses ion channel activity, exerts a crucial role in the morphogenesis of virions (10–12).

Autophagy is a process by which endogenous and foreign cytoplasmic components are encapsulated in double-membrane vesicles and transported to the lysosome for degradation. It exerts a vital role in eliminating misfolded or aggregated proteins, damaged organelles, and intracellular pathogens (13–15). As part of the innate immune response, autophagic elimination of foreign components is a selective process. This process requires the involvement of specialized cargo receptors as a link between the labeled components and the autophagic machinery (16, 17). Autophagy has been found to regulate the proliferation of many viruses. For example, by inducing autophagosome formation, the influenza A virus accelerates viral replication, weakens antigenicity, escapes host immunity, and elicits autophagic cell death (18). It is indicated that HNRNPA1 ubiquitinates PEDV N protein through MARCH8, an E3 ubiquitination ligase, thus recognized by CALCOCO2, a cargo receptor, for transport to autolysosomes (2). Moreover, it was demonstrated that SADS-CoV infection activates the autophagy pathway, facilitating its replication in Vero E6 and IPI-FX cells (19).

POLM (also designated Pol μ) represents a mammalian X-family DNA polymerase. In this family, POLM is the sole DNA polymerase capable of both template-dependent and template-independent double-strand break (DSB) repair, as well as the extension of the unpaired 3′-end in the nonhomologous end-joining (NHEJ) pathway (20, 21). POLM has been reported as the primary DNA polymerase fulfilling the fill-in function for 5′ overhangs of staggered Cas9 cleavage ends (22). POLM is required for the normal development of retina, which also supports the DSB generation and their NHEJ-mediated repair as the genuine processes implicated in neurodevelopment (23). However, the impact of POLM on virus proliferation and how to regulate virus proliferation are not clear.

This work explored the anti-viral effect of POLM upon PEDV infection. The results showed that FOXA1 acted as the transcriptional regulator of POLM expression, which exhibited PEDV inhibition. In addition, we also observed that POLM recruited MARCH8, an E3 ubiquitination ligase, to catalyze the viral N, S2, and M protein ubiquitination. The ubiquitinated viral proteins were then recognized and transferred to autophagosomes for p62-mediated selective degradation.

## RESULTS

### Transcription factor FOXA1 upregulates POLM expression during PEDV infection

**Fig 1:**
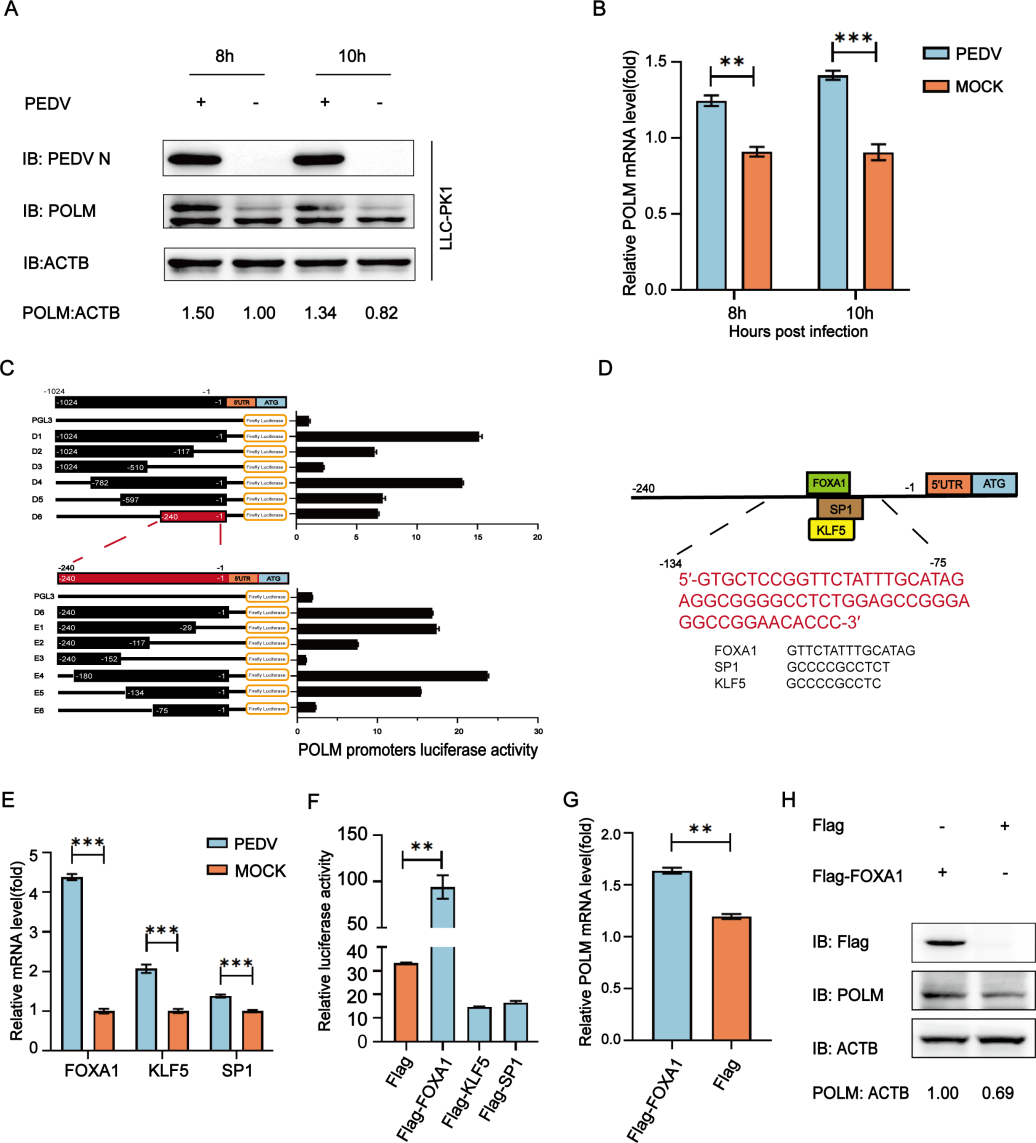
The transcription factor FOXA1 upregulates POLM expression after PEDV infection. (**A and B**) After 8 and 10 h of PEDV challenge (MOI = 0.1), the LLC-PK1 cells were harvested for Western blotting and qRT-PCR. (**C**) Truncated POLM promoter constructs (−1,024 to −1) comprising pGL3-Basic luciferase vector were transfected into HEK 293T cells for the luciferase activity determination. (**D**) JASPAR vertebrate database (http://jaspar.genereg.net) was utilized for predicting regulatory elements on the POLM promoter region. (**E**) After 10 h of PEDV challenge (MOI = 0.1), the LLC-PK1 cells were analyzed for mRNA levels of FOXA1, SP1, and KLF5 by qRT-PCR. (**F**) After transfecting HEK 293T cells with pRL-TK-luc vector, POLM promoter-driven luciferase vector, and Flag-tagged putative transcription factor (Flag-SP1, Flag-FOXA1, or Flag-KLF5)-encoding plasmids, the dual-luciferase activity was determined. (**G**) After transfecting a Flag-FOXA1-encoding plasmid into LLC-PK1 cells, the transcriptional expression of POLM was quantified by qRT-PCR. (**H**) After transfecting a Flag-FOXA1-encoding plasmid into LLC-PK1 cells, the expression of POLM was quantified by Western blot. *P < 0.05; **P < 0.01; ***P < 0.001.

The POLM protein was found to be immunoprecipitated with PEDV N by co-immunoprecipitation (Co-IP) and mass spectrometry assays. To investigate the POLM expression during infection, LLC-PK1 cells were infected with PEDV (0.1 multiplicity of infection [MOI]). Western blot and quantitative reverse transcription PCR (qRT-PCR) (Table 1 ) results suggested elevations in both the POLM protein and mRNA levels during PEDV infection (Fig. 1A and B), which suggested that PEDV infection increased POLM expression in LLC-PK1 cells. For exploration into the mechanism of upregulated expression of the POLM protein during PEDV infection, we cloned 1,024 bp sequences of POLM promoter and then truncated the promoter sequences into luciferase vector (D1–D6) to test their ability to induce luciferase levels in HEK 293T cells. It was indicated that the truncated sequences containing nucleotides from −240 to −1 induced the luciferase expression, suggesting that the POLM core promoter region lies at position −240. To further identify the boundaries for minimal POLM promoter, the D6 region was truncated and then cloned into a luciferase vector for examining luciferase activity induced by it. The core region of POLM promoter was found to span positions −134 to −75 (Fig. 1C).

**TABLE 1:** Sequences of primers and siRNAs applied in this study

Purpose	Name	Sequence (5′−3′)
Real-time PCR primers	PEDV N forward	GAGGGTGTTTTCTGGGTTG
	PEDV N reverse	CGTGAAGTAGGAGGTGTGTTAG
	POLM forward	CGGCGCTGTTAGACGTAAGC
	POLM reverse	GAGAGGCTGGCATTGTGGTG
	pGAPDH forward	ATGGATGACGATATTGCTGCGCTC
	pGAPDH reverse	TTCTCACGGTTGGCTTTGG
siRNA sequences	si-POLM sense	GCCUUUGAGAUGAGUCUCUTT
	si-POLM antisense	AAGCCTTTGAGATGAGTCTCT
	NC sense	UUCUCCGAACGUGUCACGUTT
	NC antisense	ACGUGACACGUUCGGAGAATT
	si-MARCH8 sense	GCCUAUAAUAGAGUGAUCUTT
	si-MARCH8 antisense	AGAUCACUCUAUUAUAGGCTT
	si-p62 sense	GGUGCAAAGAUCUCUGUAUTT
	si-p62 antisense	AUACAGAGAUCUUUGCACCTT

For the POLM promoter, its underlying transcriptional factor-binding sites (TFBSs) were predicted by the JASPAR database (http://jaspar.genereg.net/). We found that the FOXA1-, SP1-, and KLF5-binding sites were conserved in this minimal POLM promoter region (Fig. 1D). Upon analysis of qRT-PCR results for all predicted TFBS, it was observed that mRNA levels of the entire predicted transcription factors were upregulated in PEDV-challenged LLC-PK1 cells at 10 h (Fig. 1E), consistent with the endogenous expression trend of POLM mRNA in PEDV infection. Subsequently, we conducted a luciferase reporter assay and found that overexpression of FOXA1 significantly stimulated POLM luciferase activity (Fig. 1F). Furthermore, LLC-PK1 cells were co-transfected with Flag-FOXA1 or Flag, and the POLM mRNA levels were quantified to confirm that FOXA1 induced POLM expression (Fig. 1G). LLC-PK1 cells were co-transfected with Flag-FOXA1 or Flag, and the POLM protein levels were quantified to confirm that FOXA1 induced POLM expression (Fig. 1H). The above results demonstrate that the POLM expression was affected by FOXA1 in PEDV-challenged cells.

### POLM suppresses PEDV replication in LLC-PK1 cells

**Fig 2:**
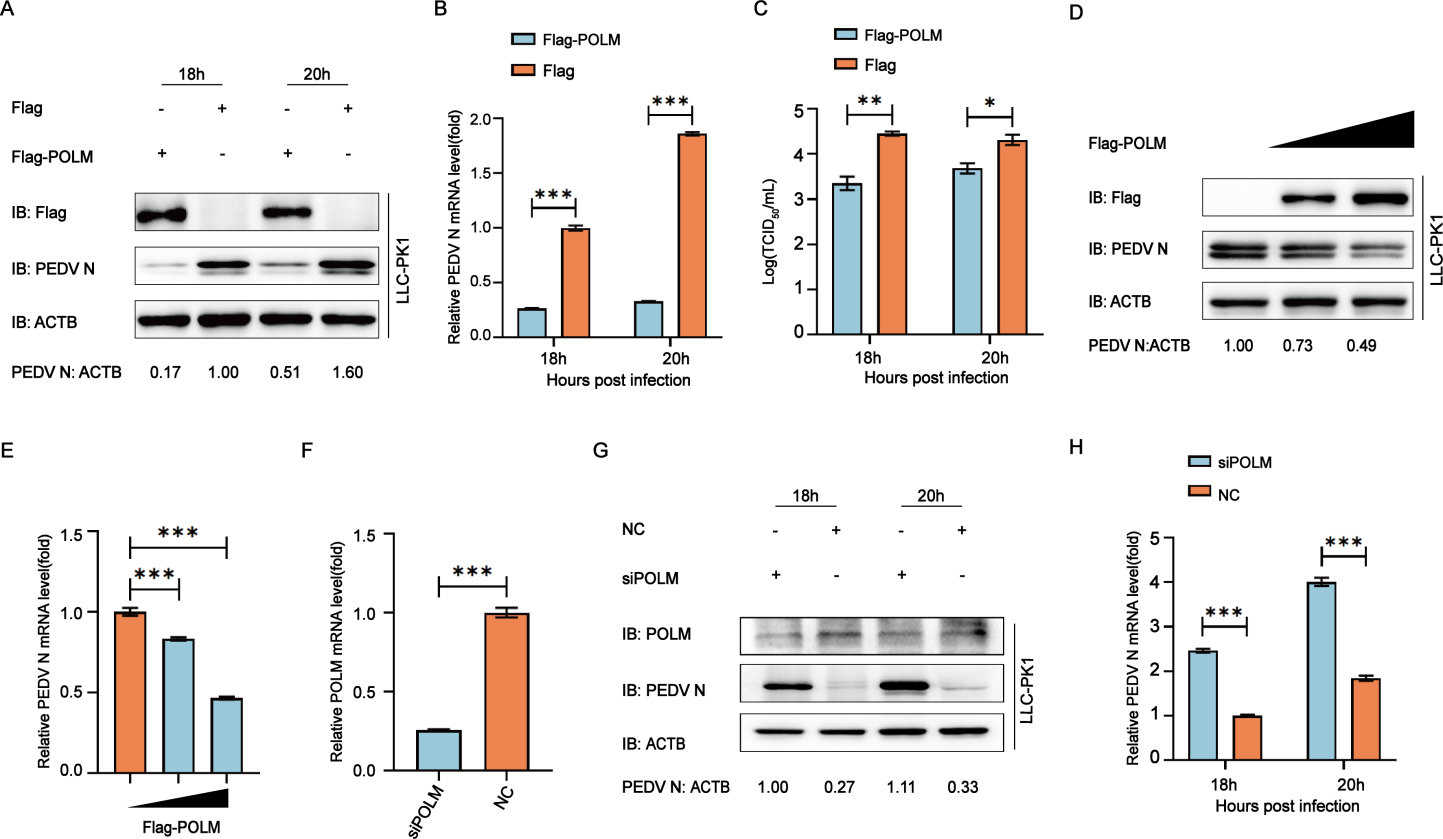
POLM inhibits the proliferation of PEDV. (**A and B**) After transfection with Flag-POLM or Flag-encoded plasmids, the LLC-PK1 cells were exposed to 0.01 MOI PEDV and sampled at 18 and 24 h post-infection for assessing the PEDV N protein and mRNA levels through Western blotting combined with qRT-PCR. (**C**) The supernatant of the culture harvested in panel **A** was utilized for the determination of viral titers at TCID_50_. (**D and E**) After transfecting different doses of Flag-POLM plasmids, the LLC-PK1 cells were subjected to PEDV challenge (MOI = 0.01) and subsequently harvested for Western blotting and qRT-PCR assays. (**F**) qRT-PCR was conducted for the interference efficiency characterization of POLM small interfering RNA (siRNA). (**G and H**) After transfecting POLM siRNA or negative control siRNA, the LLC-PK1 cells were subjected to PEDV challenge (MOI = 0.01), followed by collection of the cells and supernatants at 18 and 20 h after PEDV infection to detect PEDV N protein and mRNA levels *P < 0.05; **P < 0.01; ***P < 0.001.

To explore POLM’s function in PEDV infection, LLC-PK1 cells were transfected with POLM plasmid (Flag-POLM) and subsequently exposed to 0.01 MOI PEDV. Cells and supernatants were collected after 18 and 20 h of PEDV infection, and the PEDV N protein expression was evaluated through Western blot, qRT-PCR, and 50% tissue culture infective dose (TCID_50_) assay. We revealed that the viral protein, mRNA, and viral load levels were significantly lower compared to the empty vector control (Fig. 2A through C). Furthermore, as the quantity of POLM plasmid increased, the level of PEDV N decreased accordingly (Fig. 2D and E). LLC-PK1 cells were transfected with small interfering RNAs targeting the mRNA sequence of POLM and collected after 18 and 20 h of PEDV infection. We found that decreasing the POLM expression significantly facilitated the PEDV proliferation (Fig. 2F through H). These findings suggested the inhibition of PEDV replication by the host factor POLM in LLC-PK1 cells.

### POLM interacts with PEDV N, S2, and M proteins

**Fig 3:**
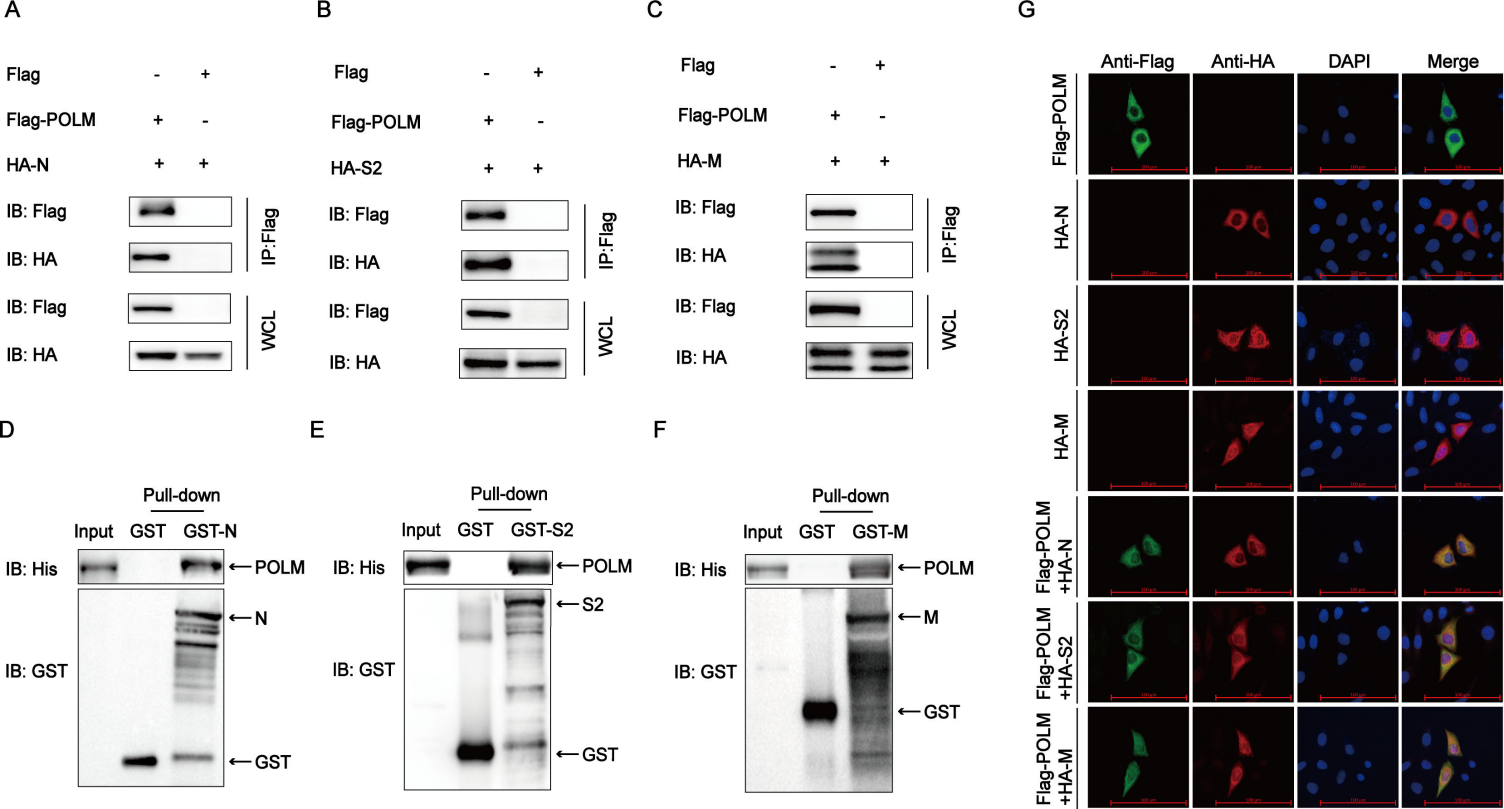
POLM interacts with PEDV N, S2, and M proteins. (**A–C**) After transfecting HEK 293T cells with Flag-POLM and HA-N, HA-S2, and HA-M plasmids, the protein interactions were assayed by Co-IP. (**D–F**) After denotation in strain BL21 (DE3), GST pull-down assay was conducted to study the interactions of POLM with GST-N, GST-S2, and GST-M. (**G**) After transfection of HeLa cells with plasmids encoding HA-N, HA-S2, HA-M, and Flag-POLM, a designated primary antibody was used for labeling the HA-N, HA-S2, HA-M, and Flag-POLM. Nuclear staining was performed with 4′,6-diamidino-2-phenylindole, followed by observation of fluorescence signal by confocal immunofluorescent microscopy. Scale bars: 100 µm.

To clarify the mechanism through which POLM inhibits PEDV replication, we initially conducted a Co-IP assay by co-transfecting HEK 293T cells with POLM-coding plasmids and PEDV N, S1, S2, M, and E proteins. The Co-IP result exhibited a correlation between POLM and the PEDV N, S2, and M proteins (Fig. 3A through C). A GST pull-down experiment confirmed that POLM was associated directly with the PEDV N protein, PEDV S2 protein, and PEDV M protein (Fig. 3D through F). To investigate the POLM co-localization with the aforementioned viral structural proteins at a subcellular level, we co-transfected HeLa cells with Flag-POLM and HA-N, HA-S2, and HA-M plasmids, and protein localization was examined by confocal immunofluorescence assay. Cytoplasmic co-localization of POLM and N, S2, and M proteins was observed (Fig. 3G). Collectively, these data indicated the interaction of POLM with PEDV N, S2, and M proteins.

### POLM degrades PEDV N, S2, and M proteins by autophagy

**Fig 4:**
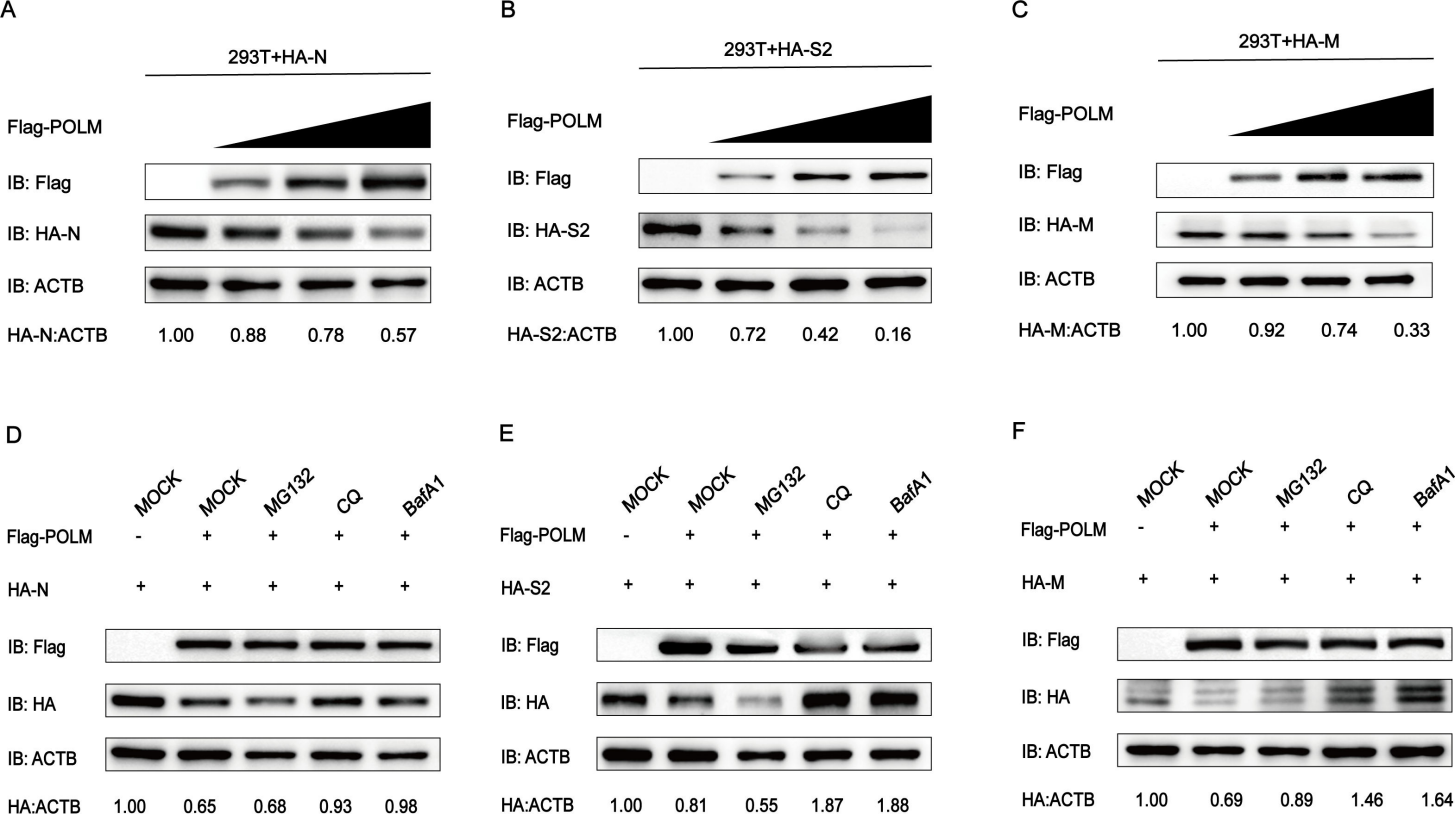
POLM degrades PEDV N, S2, andbM proteins via the autophagy pathway. (**A–C**) After transfecting HEK 293T cells with varying doses of Flag-POLM and HA-N, HA-S2, and HA-M, respectively, Western blotting was conducted to observe the N, S2, and M expressions. (**D–F**) After co-transfection with plasmids encoding Flag-POLM and HA-N, HA-S2, or HA-M, respectively, the HEK 293T cells were treated using autophagy and ubiquitination inhibitors for the protein expression observation via Western blot.

Considering that POLM has been demonstrated to suppress the PEDV replication and to interact with N, S2, and M proteins of this virus, its role in the N, S2, and M protein expression was investigated first. After co-transfecting Flag-POLM and HA-N, HA-S2, and HA-M plasmids into HEK 293T cells, Western blot was conducted to assess the N, S2, and M protein levels. Drastic dose-responsive decreases in the N, S2, and M expressions were observed in POLM-transfected cells (Fig. 4A through C). Degradation of eukaryotic proteins primarily involves the ubiquitin-proteasome and autophagy-lysosome axes. To determine which protein degradation pathway was involved in the POLM degradation of N, S2, and M, we co-transfected Flag-POLM and HA-N, HA-S2, and HA-M plasmids into HEK 293T cells. Simultaneously, inhibitors of autophagy-lysosome (CQ and BafA1) and ubiquitinated proteasome (MG132) were used for the cell treatment. Our results suggested that the N, S2, and M protein degradation by POLM was inhibited by BafA1 and CQ (Fig. 4D through F), not by MG132, indicating that POLM may degrade PEDV N, S2, and M proteins through the autophagy pathway.

### POLM interacts with MARCH8 and p62

**Fig 5:**
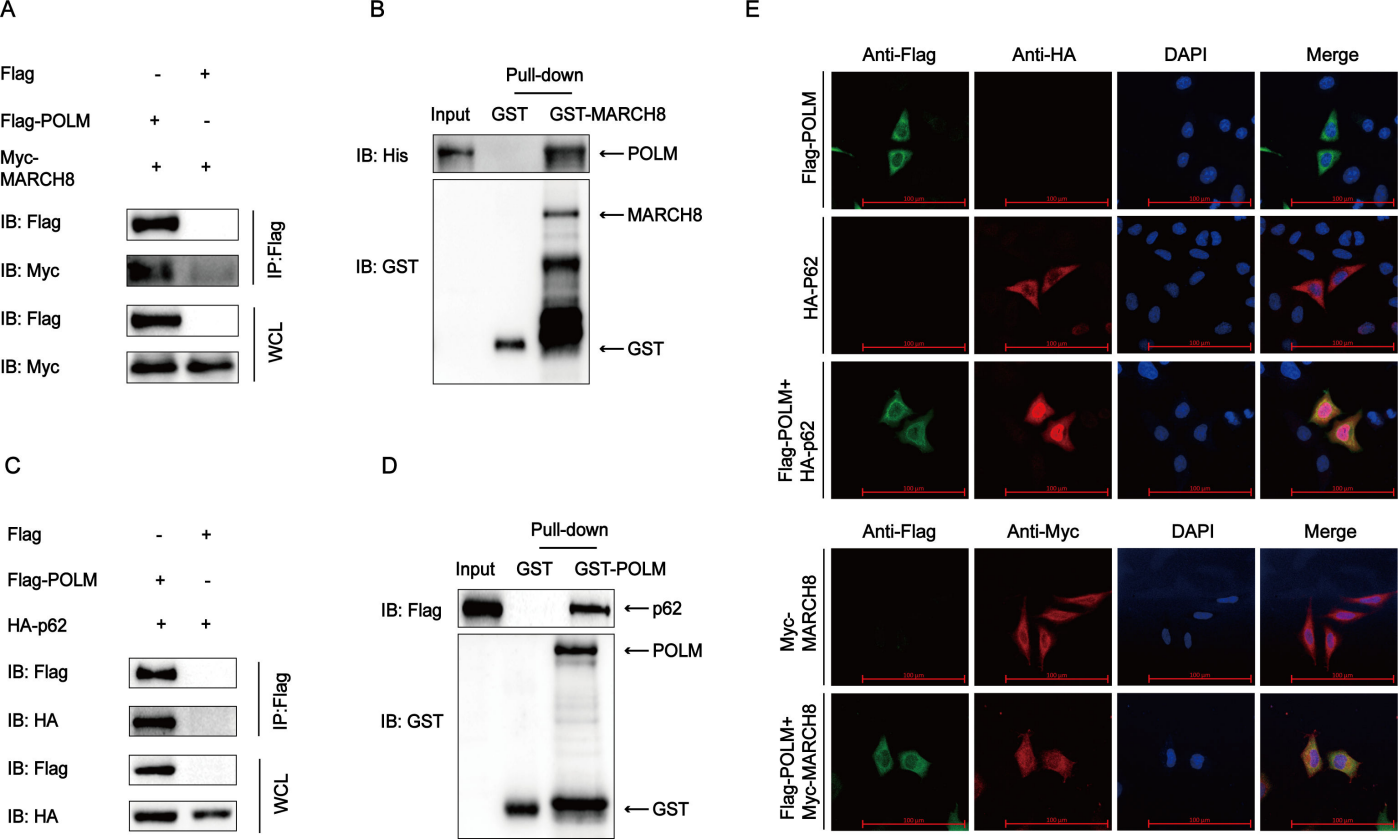
POLM interacts with MARCH8 and p62. (**A and C**) After co-transfection of HEK 293T cells with Flag-POLM and Myc-MARCH8 or HA-p62, their interactions were analyzed by Co-IP. (**B and D**) GST affinity-isolation assay was carried out to assess GST-MARCH8 and POLM or GST-POLM and p62. (**E**) After transfecting Flag-POLM and Myc-MARCH8 or HA-p62, respectively, the HeLa cells were labeled using the corresponding antibodies for confocal immunofluorescence microscopic observation. Scale bars: 100 µm.

The autophagy protein degradation pathway requires the E3 ubiquitination ligases and cargo receptors. After the ubiquitination of substrate protein by E3 ubiquitination ligases and its recognition by cargo receptors, the ubiquitinated substrate protein was delivered to the autophagosome by cargo receptors. To identify which E3 ubiquitination ligase was implicated in the PEDV structural protein degradation by POLM, the HEK 293T cells were co-transfected with Flag-POLM and a range of common E3 ubiquitination ligase plasmids (including MARCH8 and STUB1). Co-IP assay revealed the interplay of POLM with MARCH8 (Fig. 5A). Besides, GST pull-down assays demonstrated that GST-MARCH8 interplayed with POLM, while individual GST proteins did not (Fig. 5B), suggesting that MARCH8 interplayed directly with POLM. To identify which cargo receptor was implicated in the degradation, Flag-POLM, along with some commonly used cargo receptors, was transfected into HEK 293T cells. We found that POLM interacted with p62 (Fig. 5C); this interaction was also confirmed by GST pull-down and confocal microscopy assays (Fig. 5D and E). Collectively, these data demonstrated the interaction of POLM with MARCH8 and p62.

### PEDV replication can be hindered by POLM through the MARCH8-p62-autophagosome pathway

**Fig 6:**
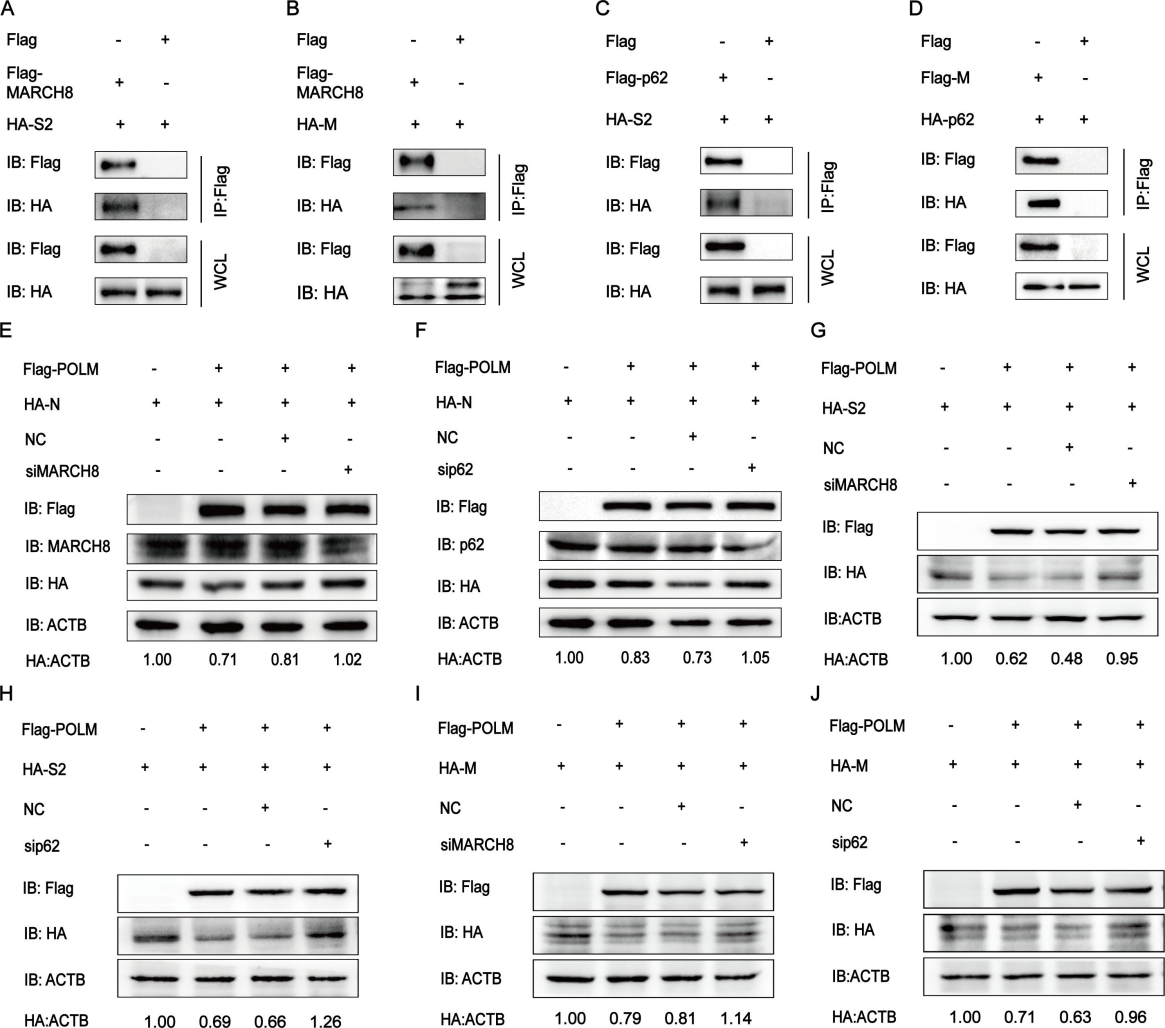
POLM degrades PEDV structural proteins via the MARCH8-p62-autophagosome axis. (**A and B**) After co-transfecting Flag-MARCH8 and HA-S2 or HA-M plasmids into HEK 293T cells, the protein interactions were assayed by Co-IP. (**C and D**) After co-transfecting plasmids coding p62 and S2 or M into HEK 293T cells, the protein interactions were assayed by Co-IP. (**E and F**) HEK 293T cells were transfected with plasmids encoding Flag-POLM and HA-N, as well as siMARCH8 or sip62 for subsequent analysis via Western blot. (**G and H**) HEK 293T cells were transfected with plasmids encoding Flag-POLM and HA-S2, as well as siMARCH8 or sip62 for subsequent analysis via Western blot. (**I and J**) HEK 293T cells were transfected with plasmids encoding Flag-POLM and HA-M, as well as siMARCH8 or sip62 for subsequent analysis via Western blot.

In numerous forms of selective autophagy, protein ubiquitination functions as a cargo recognition biomarker and a process initiation signal, with ubiquitinated proteins exerting a role in the recruitment of specific autophagic effectors to facilitate autophagy (24). Previous results have indicated that the PEDV N protein interacts with MACH8 and p62 (25, 26). To confirm the interaction between the substrate proteins (S2 and M) and MARCH8 or p62, we co-transfected Flag-MARCH8 and HA-S2 or HA-M plasmids, respectively, into HEK 293T cells. According to our Co-IP result, MARCH8 interacted with S2 and M proteins (Fig. 6A and B). Furthermore, S2 and M have been identified as the interaction with p62 through the Co-IP assay (Fig. 6C and D). To ascertain the roles of p62 and MARCH8 in POLM-induced PEDV N, S2, and M protein degradation via autophagy pathway, siMARCH8 or sip62 was used to cut off the MARCH8-p62-autophagosome pathway and detected N, S2, and M proteins degraded by POLM (Table 1). Our findings showed that the decrease in p62 or MARCH8 protein expression remarkably inhibited the N, S2, and M protein degradation induced by POLM (Fig. 6E through J), suggesting a drastically weakened ability of POLM to degrade N, S2, and M proteins upon the autophagy pathway interruption. The above results implied autophagic degradation of PEDV N, S2, and M proteins via the POLM-MARCH8-p62-autophagosome axis.

## DISCUSSION

**Fig 7:**
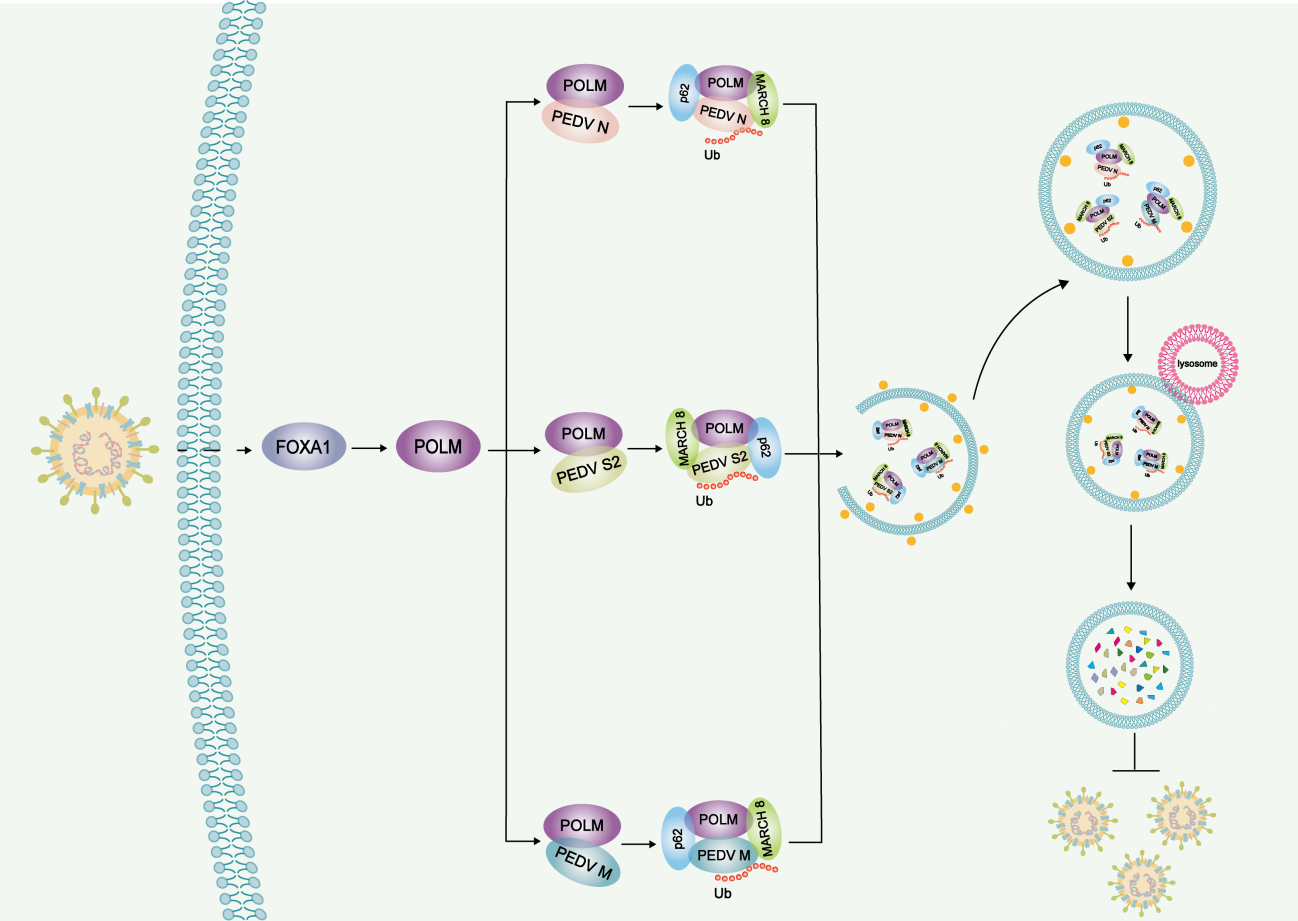
Anti-viral mechanisms of POLM. During PEDV infection, the transcription factor FOXA1 can bind to the POLM promoter and regulate the expression of POLM. POLM ubiquitinates PEDV N, S2, and M proteins by recruiting the E3 ubiquitination ligase MARCH8. Next, the ubiquitinated proteins are recognized by the cargo receptor p62, translocated to the lysosome and degraded by the autophagy, which can therefore inhibit PEDV proliferation.

PEDV is a threat to the swine industry causing huge economic losses because no effective vaccines are currently available. Therefore, it is urgent to strengthen the research on the interaction between PEDV and hosts. This study explored the modification of potential host anti-viral factors in response to PEDV infection, as well as its regulatory role in the PEDV replication process. We found that the newly discovered host anti-viral factor POLM could be regulated by FOXA1 in the infection process with PEDV. Furthermore, we unraveled the anti-PEDV replication mechanism of POLM, which was realized by promoting the viral N, S2, and M protein degradation via the POLM-MARCH8-p62 autophagosome pathway (Fig. 7).

POLM belongs to the DNA polymerase X family and contains an N-terminal BRCT domain that interacts with X4L4, Ku, and other NHEJ proteins alongside DNA substrates, aiming to facilitate POLM binding to DNA and improve the primer extension efficiency (27, 28). There have been no reports of POLM with viruses, and we have found its anti-viral function in PEDV replication and identified the core POLM promoter region to span −134 to −75. We observed and verified the POLM upregulation after PEDV infection through the core promoter FOXA1. In LLC-PK1 cells, overexpression of POLM inhibited PEDV proliferation at various time points. Co-immunoprecipitation of POLM with N, S2, and M proteins of PEDV was noted. These findings suggest that POLM represents a potential target for managing PEDV infection.

As a vital and conserved self-balancing mechanism, autophagy exerts a crucial role in degrading cytoplasmic proteins and organelles, serving as an early defense against viral infection (29–31). During autophagy, autophagosomes are immobilized in the cytoplasm and subsequently fuse with lysosomes, enabling the transport of substances to the organelle lumen, where they are broken down (32). In recent years, we have indicated mounting evidence indicating the potential of the autophagy-lysosomal pathway for curbing the proliferation of viruses. The host proteins FUBP3, PRPF19, and PTBP1 have been demonstrated to impede the replication of PEDV through the autophagy-lysosomal pathway (33–35). PGAM5 proteins utilize the autophagy-lysosomal pathway to inhibit porcine delta coronavirus replication (36). In this study, we demonstrated that POLM was capable of impeding PEDV replication by facilitating the viral N, S2, and M protein degradation through the autophagy-lysosomal pathway.

Autophagy receptors serve as a conduit between ubiquitination and the autophagy-lysosome system, exemplified by NDP52 and p62 (37, 38). During the selective autophagy process, E3 ubiquitination ligases are capable of ubiquitinating substrate proteins. After recognition by cargo receptors, the ubiquitinated substrate proteins are then imported into lysosomes for degradation (39). Our results demonstrated an interaction between the host protein POLM and the cargo receptor p62 and the E3 ubiquitin ligase MARCH8. Interference with p62 or MARCH8 expression resulted in alterations in the PEDV N, S2, and M protein degradation by POLM. These findings suggest that the overexpression of POLM recruits MARCH8, which acts as a ubiquitination catalyst for N, S2, and M proteins of PEDV. These are subsequently recognized by the cargo receptor p62, therefore facilitating their translocation to the lysosome for degradation.

In conclusion, we demonstrate that POLM suppresses PEDV replication by degrading the viral structural (N, S2, and M) proteins. The transcription factor FOXA1 binds to the core POLM promoter region to upregulate the POLM expression during PEDV infection. The inhibition of PEDV proliferation by POLM is achieved through the degradation of PEDV N, S2, and M proteins via the MARCH8 and p62-mediated autophagic lysosomal pathway. In this study, we offer evidence for a novel anti-viral infection mechanism mediated by POLM. The host protective role of POLM against PEDV infection probably lays the basis for a potential innovative treatment strategy.

## MATERIALS AND METHODS

### Antibodies and reagents

The anti-POLM antibody (DF10080) utilized in this study was provided by Affinity. Furthermore, we used an anti-GST antibody (HRP-66001), anti-ACTB/β-actin antibody (66009–1-Ig), along with horseradish peroxidase-conjugated anti-mouse and anti-rabbit IgG antibodies (SA00001–1 and SA00001–2) from Proteintech. Reagents, including bafilomycin A1 (BafA1, 54645), MG132 (M7449), and chloroquine phosphate (CQ, PHR1258), and antibodies like HA-tag (H6908), anti-Myc-tag (2276S), and anti-FLAG M2 (F1804) were Sigma-Aldrich products. The anti-PEDV N protein antibody was from our laboratory.

### Cell culture and virus

Dulbecco’s modified Eagle medium (D6429, Sigma-Aldrich) containing 10% fetal bovine serum (F0193, Sigma-Aldrich) was used to culture HEK 293T cells (CRL-11268, American Type Culture Collection), while MEM (11095080, Gibco) was utilized to culture LLC-PK1 cells provided by Rui Luo from Huazhong Agricultural University (Wuhan, China). Both cell lines were cultured in a 5% CO_2_ atmosphere at 37°C. The PEDV strain employed in this study was the laboratory-isolated PEDV variant JS-2013 (MH910099).

### Plasmids and transfection

The vectors and label proteins used in this study, namely, Flag, pGL3-Basic, PXJ41-HA, PXJ41-Myc, Pcold-TF, and Pcold-GST, were obtained from the laboratory depository. The exogenous DNA is inserted into the vector through the use of enzymatic ligation and homologous recombination. Plasmids were subjected to homologous recombination cloning through a one-step cloning kit (C112–02, Vazyme Biotech). Upon reaching cellular confluency of 80%–90%, the plasmids were transfected by applying the Lipofectamine 3000 reagent (L300015, Invitrogen). Interfering RNA, which was synthesized at GenePharma (Shanghai), was transfected following the protocol of Lipofectamine RNAiMAX (13778150, Invitrogen).

### qRT-PCR

Extraction of total RNA was performed through the FastPure Viral DNA/RNA Mini Kit (RC311, Vazyme Biotech) or the FastPure Cell/Tissue Total RNA Isolation Kit (RC101–01, Vazyme Biotech). Subsequently, the extracted total RNA was reversely transcribed into cDNA via the HiScript III RT SuperMix for qPCR (+gDNA wiper) (R323–01, Vazyme Biotech). Then, qRT-PCR was conducted following the protocol of SYBR premix Ex Taq (q711–03, Vazyme Biotech).

### Western blot assay

After rinsing in cold phosphate-buffered saline, the cells were subjected to 5 min of lysis on ice by RIPA buffer (89901, Thermo Fisher Scientific) including either protease inhibitor (SB-WB016, Share-bio). Then, the samples were boiled in a proper volume of 5× SDS-PAGE loading buffer (SB-PR037, Share-bio) for 10 min and electrophoretically separated. Subsequently, the proteins were shifted onto nitrocellulose blotting membranes (10,600,001; GE Healthcare) and incubated with the designated primary antibody and secondary antibody. Finally, protein assays were conducted with an enhanced chemiluminescence chemiluminescent substrate (SB-WB012, Share-bio).

### Co-IP assay

Following 24 h of plasmid transfection, the cells were collected using a protease inhibitor-containing lysis buffer. For protein isolation from the cells, the anti-Flag antibody was incubated with protein G (Dynabeads, 10004D; Thermo Fisher Scientific), followed by rinsing of the Dynabeads in 0.02% phosphate buffered saline Tween (PBST). The subsequent step was elution of proteins in a pH 2.8 glycine buffer. Next, the eluted proteins were subsequently explored by Western blot assay.

### GST affinity isolation assay

After cloning full-length sequences of PEDV *N*, *S2*, *M*, *POLM*, *p62*, and *MARCH8* into the Pcold-GST or Pcold-TF vectors, the BL21-competent cells (C504–03, Vazyme Biotech) were used for the plasmid transformation. For the GST affinity-isolation assay, recombinant proteins were expressed and purified following the protocol of GST Protein Interaction Pull-Down Kit (21516, Thermo).

### Immunofluorescence assay

After immobilizing in 4% paraformaldehyde (P6148), the cell samples were permeabilized with 0.1% Triton X-100 (9284, both Sigma-Aldrich), followed by 60 min of incubation with 3% bovine serum albumin-diluted primary and secondary antibodies at 37°C. Finally, cell observation was performed by confocal immunofluorescence microscopy (Carl Zeiss, Germany).

### Luciferase reporter assay

The luciferase reporter gene, TK plasmid, and target gene were co-transfected into HEK 293T cells. The cells were harvested 24 h later for the luciferase activity measurement following the protocol of the Dual-Glo luciferase assay system (L101, Vazyme Biotech).

### Statistical analysis

Intergroup comparison was made through two-sided Student’s *t*-test with the aid of Prism version 5 (GraphPad Software). Significant differences were indicated by *P < 0.05, **P < 0.01, and ***P < 0.001. All the values presented are means from triplicate experiments.

## Data Availability

All data are contained within the article.
